# Positive Predictive Value of Myelin Oligodendrocyte Glycoprotein Autoantibody Testing

**DOI:** 10.1001/jamaneurol.2021.0912

**Published:** 2021-04-26

**Authors:** Elia Sechi, Marina Buciuc, Sean J. Pittock, John J. Chen, James P. Fryer, Sarah M. Jenkins, Adrian Budhram, Brian G. Weinshenker, A. Sebastian Lopez-Chiriboga, Jan-Mendelt Tillema, Andrew McKeon, John R. Mills, W. Oliver Tobin, Eoin P. Flanagan

**Affiliations:** 1 Department of Neurology, Mayo Clinic College of Medicine and Science, Rochester, Minnesota; 2 Department of Medical, Surgical and Experimental Sciences, University of Sassari, Sassari, Italy; 3 Department of Laboratory Medicine and Pathology, Mayo Clinic College of Medicine and Science, Rochester, Minnesota; 4 Department of Ophthalmology, Mayo Clinic College of Medicine and Science, Rochester, Minnesota; 5 Department of Health Sciences Research, Mayo Clinic College of Medicine and Science, Rochester, Minnesota; 6 Department of Clinical Neurological Sciences, London Health Sciences Centre, Western University, London, Ontario, Canada; 7 Department of Neurology, Mayo Clinic, Jacksonville, Florida

## Abstract

**Importance:**

Myelin oligodendrocyte glycoprotein-IgG1–associated disorder (MOGAD) is a distinct central nervous system–demyelinating disease. Positive results on MOG-IgG1 testing by live cell-based assays can confirm a MOGAD diagnosis, but false-positive results may occur.

**Objective:**

To determine the positive predictive value (PPV) of MOG-IgG1 testing in a tertiary referral center.

**Design, Setting, and Participants:**

This diagnostic study was conducted over 2 years, from January 1, 2018, through December 31, 2019. Patients in the Mayo Clinic who were consecutively tested for MOG-IgG1 by live cell-based flow cytometry during their diagnostic workup were included. Patients without research authorization were excluded.

**Main Outcomes and Measures:**

Medical records of patients who were tested were initially reviewed by 2 investigators blinded to MOG-IgG1 serostatus, and pretest probability was classified as high or low (suggestive of MOGAD or not). Testing of MOG-IgG1 used a live-cell fluorescence-activated cell-sorting assay; an IgG binding index value of 2.5 or more with an end titer of 1:20 or more was considered positive. Cases positive for MOG-IgG1 were independently designated by 2 neurologists as true-positive or false-positive results at last follow-up, based on current international recommendations on diagnosis or identification of alternative diagnoses; consensus was reached for cases in which disagreement existed.

**Results:**

A total of 1617 patients were tested, and 357 were excluded. Among 1260 included patients tested over 2 years, the median (range) age at testing was 46 (0-98) years, and 792 patients were female (62.9%). A total of 92 of 1260 (7.3%) were positive for MOG-IgG1. Twenty-six results (28%) were designated as false positive by the 2 raters, with an overall agreement on 91 of 92 cases (99%) for true and false positivity. Alternative diagnoses included multiple sclerosis (n = 11), infarction (n = 3), B_12_ deficiency (n = 2), neoplasia (n = 2), genetically confirmed adrenomyeloneuropathy (n = 1), and other conditions (n = 7). The overall PPV (number of true-positive results/total positive results) was 72% (95% CI, 62%-80%) and titer dependent (PPVs: 1:1000, 100%; 1:100, 82%; 1:20-40, 51%). The median titer was higher with true-positive results (1:100 [range, 1:20-1:10000]) than false-positive results (1:40 [range, 1:20-1:100]; *P* < .001). The PPV was higher for children (94% [95% CI, 72%-99%]) vs adults (67% [95% CI, 56%-77%]) and patients with high pretest probability (85% [95% CI, 76%-92%]) vs low pretest probability (12% [95% CI, 3%-34%]). The specificity of MOG-IgG1 testing was 97.8%.

**Conclusions and Relevance:**

This study confirms MOG-IgG1 as a highly specific biomarker for MOGAD, but when using a cutoff of 1:20, it has a low PPV of 72%. Caution is advised in the interpretation of low titers among patients with atypical phenotypes, because ordering MOG-IgG1 in low pretest probability situations will increase the proportion of false-positive results.

## Key Points

**Question:**

What is the positive predictive value of myelin oligodendrocyte glycoprotein (MOG)–IgG1 testing in a clinical setting?

**Findings:**

Of 1260 consecutive patients tested for MOG-IgG1 at the Mayo Clinic over 2 years, 92 (7.3%) were positive, 26 (28%) of whom had their results independently designated as false positive by 2 neurologists. The positive predictive value was 72% and varied with autoantibody titer (≥1:1000, 100%; 1:100, 82%; 1:20-40, 51%) and clinical–magnetic resonance imaging phenotypes at testing (pretest probability: high, 85%; low, 12%).

**Meaning:**

False-positive MOG-IgG1 results are encountered in clinical practice; caution is advised before assigning a MOG-IgG1–associated disorder diagnosis in patients with low-titer positive results and atypical phenotypes.

## Teaser

This diagnostic study seeks to determine the positive predictive value of myelin oligodendrocyte glycoprotein–IgG1 testing in patients in a single tertiary referral center.

## Introduction

Myelin oligodendrocyte glycoprotein (MOG)–IgG1–associated disorder (MOGAD) is a distinct central nervous system (CNS)–demyelinating disease characterized by attacks of optic neuritis, myelitis, brain or brainstem inflammation, or combinations thereof.^1^ Seropositivity for MOG-IgG1 confirms the diagnosis with a compatible clinical and radiologic phenotype,^2^ with important therapeutic and prognostic implications.^3,4^ International comparative studies of different MOG-IgG1 assays have shown that live cell–based assays yield the highest specificity for MOGAD.^5,6^ However, MOGAD is rare, and indiscriminate testing for MOG-IgG1 may lead to false-positive results despite high specificity.^7^ The positive predictive value (PPV), which provides the likelihood that a positive test result is truly positive for the disease of interest, is arguably of greater clinical utility. Studies examining the PPV of MOG-IgG1 testing in clinical practice are lacking yet crucial to better interpret test results. We studied the PPV of MOG-IgG1 testing in a large cohort from a tertiary referral center.

## Methods

The Mayo Clinic institutional review board approved the study. All patients provided written informed consent. Patients without research authorization were excluded.

### Study Population

We included consecutive patients who were seen at the Mayo Clinic between January 1, 2018, and December 31, 2019, and tested for MOG-IgG1 as part of routine clinical care. Details are in the eFigure in the Supplement.

### Autoantibody Testing

Testing for MOG-IgG1 was performed with a live cell–based flow cytometry or fluorescence-activated cell-sorting assay with full-length MOG in its conformational form. Serum samples were screened at 1:20 dilution, and if the IgG-binding index (IBI; a ratio of median fluorescence intensities of MOG-transfected vs MOG-nontransfected cells) was 2.5 or more, they were diluted at 1:20, 1:40, 1:100, and 10-fold thereafter to establish end-titer values (last dilution with an IBI ≥2.5; reference value, <1:20).^5^

### Pretest Probability

Medical records were initially reviewed by 2 investigators (E.S. and M.B.) blinded to MOG-IgG1 serostatus to determine demographic, clinical, magnetic resonance imaging (MRI), and cerebrospinal fluid characteristics of patients at the time of testing. The pretest probability for MOGAD was considered high with acute attacks (nadir ≤1 month) of (1) optic neuropathy, (2) myelopathy, (3) brain or brainstem demyelination, (4) unilateral cortical encephalitis, or (5) multifocal CNS demyelination.^2,8^ Patients with typical multiple sclerosis (MS) lesions on brain MRI^9,10^ or other phenotypes were designated as having low pretest probability.

### True-Positive vs False-Positive Assessment

The medical records and MRIs of individuals positive for MOG-IgG1 at last follow-up were independently reviewed by 2 neurologists (E.S. and E.P.F.), and true-positive results were defined per current international recommendations on diagnosis^2^ or more recently recognized unilateral cortical encephalitis syndrome.^8^ Alternative diagnoses or phenotypes inconsistent with MOGAD were designated as false-positive results. Diagnoses of MS were based on the revised McDonald criteria,^11^ including typical MS lesions on MRI.^9^ Consensus was reached for cases in which disagreement existed.

### Statistics

Continuous and categorical variables were compared using Wilcoxon rank sum and Fisher exact tests, respectively. A *P* value less than .05 was considered statistically significant. Correlations were assessed by Spearman ρ. The PPV (true-positive results divided by total positive results) and specificity (true-negative results divided by true-negative results plus false-positive results) were reported; 95% CIs were calculated using the score method (SAS version 9.4 [SAS Institute]). Graphs were built with R version 3.6.2 (R Foundation for Statistical Computing).

## Results

A total of 1617 patients were tested, and 357 were excluded. The remaining 1260 patients were included (median [range] age at testing, 46 [0-98] years; 792 female patients [62.9%]), of whom 92 (7.3%) were positive for MOG-IgG1. Those with MOG-IgG1–positive results were younger than those with negative results (median [range] age, 36.5 [8-73] years vs 46 [0-98] years; *P* < .001). Female sex frequency was similar in the 2 groups (53 of 92 patients [58%] vs 739 of 1168 patients [63.3%]; *P* = .31).

### PPV and Specificity

**Figure 1.**
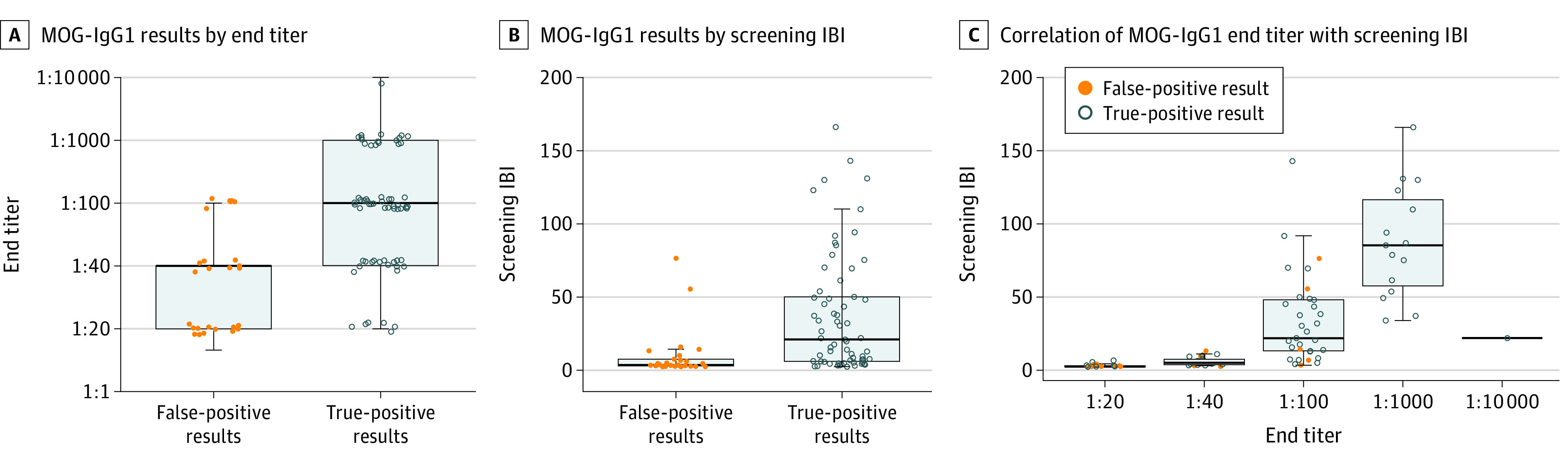
Distribution of Antibody Titers and IgG-Binding Index (IBI) Values Among True-Positive and False-Positive Myelin Oligodendrocyte Glycoprotein (MOG)–IgG1 Cases A and B, Boxplots showing the distribution of MOG-IgG1 end titers and screening IBI values, respectively, among false-positive and true-positive MOG-IgG1–associated disorder (MOGAD). Titers of 1000 or more and IBI values of 76.4 or more yield 100% specificity and positive predictive values for true MOGAD. C, Boxplot showing the correlation between MOG-IgG1 titer and IBI value (Spearman ρ, 0.86). True-positive and false-positive MOG-IgG1–positive cases in the 3 plots are displayed in gray open circles and orange dots, respectively.

The Table shows the frequencies of MOG-IgG1 positivity, false-positive results, and PPV stratified by age, antibody IBI or titer, and pretest probability. The overall PPV was 72% (95% CI, 62%-80%), and this increased with a higher MOG-IgG1 titer (≥1:1000; 100% [95% CI, 82%-100%]), higher IBI value (≥80; 100% [95% CI, 72%-100%]), lower age (<18 years; 94% [95% CI, 72%-99%]), and higher pretest probability (85% [95% CI, 76%-92%]; *P* < .001) (Table). In those with atypical phenotypes and a titer less than 1:100, the PPV was 10% (95% CI, 2%-40%), while in those with either atypical phenotypes or a titer less than 1:100, the PPV was 46% (95% CI, 33%-60%). The MOG-IgG1 titer strongly correlated with IBI value (ρ = 0.86; Figure 1). The specificity of MOG-IgG1 testing was 97.8%.

**Table.** Myelin Oligodendrocyte Glycoprotein–IgG1 (MOG-IgG1) Positivity Rate, False-Positive Rate, and Positive Predictive Value in 1260 Patients

Characteristic	No./total No. (%)		Positive predictive value, No. (%) [95%CI]
	Positive results/total tests	False-positive results/positive results	
Total cohort	92/1260 (7.3)	26/92 (28)	66/92 (72) [62-80]
Age range, y			
≥18	76/1186 (6.4)	25/76 (33)	51/76 (67) [56-77]
<18	16/74 (22)	1/16 (6)	15/16 (94) [72-99]
Screening IBI of MOG-IgG1–positive results^a^			
≥80	10/91 (11)	0	10/10 (100) [72-100]
10-79.99	37/91 (41)	6/37 (16)	31/37 (84) [69-92]
2.5-9.99	44/91 (48)	20/44 (45)	24/44 (55) [40-68]
Antibody end titer of MOG-IgG1–positive results^a^			
≥1:1000	17/91 (19)	0	17/17 (100) [82-100]
<1:1000	74/91 (81)	26/74 (35)	48/74 (65) [54-75]
1:100	33/91 (36)	6/33 (18)	27/33 (82) [66-91]
1:20-40	41/91 (45)	20/41 (49)	21/41 (51) [36-66]
Phenotype at MOG-IgG1 testing of total cohort			
Consistent with MOGAD, with high pretesting probability	75/530 (14.2)	11/75 (15)	64/75 (85) [76-92]
Multifocal central nervous system demyelination	26/91 (29)	0	26/26 (100) [87-100]
Acute brain or brainstem demyelination	6/89 (7)	4/6 (67)	2/6 (33) [10-70]
Acute optic neuropathy	31/177 (17.5)	1/31 (3)	30/31 (97) [84-99]
Acute myelopathy	11/166 (6.6)	6/11 (55)	5/11 (45) [21-72]
Longitudinally extensive T2 lesion on MRI^b^	7/65 (11)	2/7 (29)	5/7 (71) [36-92]
Unilateral cortical encephalitis	1/7 (14)	0	1/1 (100) [NA]
Atypical for MOGAD, with low pretesting probability	17/730 (2.3)	15/17 (88)	2/17 (12) [3-34]
Multiple sclerosis or clinically or radiologically isolated syndrome^c^	9/352 (2.6)	9/9 (100)	0 (0) [0-30]
Other neurological phenotypes^d^	6/307 (2.0)	4/6 (67)	2/6 (33) [10-70]^e^
Nonneurologic condition	2/71 (3)	2/2 (100)	0 (0) [NA]
Disease course at testing in those with high pretesting probability			
Monophasic	46/428 (10.7)	11/46 (24)	35/46 (76) [62-86]
Relapsing	29/102 (28.4)	0/29	29/29 (100) [88-100]

Abbreviations: IBI, IgG-binding index; MOGAD, myelin oligodendrocyte glycoprotein-IgG1–associated disorder; MRI, magnetic resonance imaging; NA, not applicable.

^a^ The exact IBI and end-titer values were not available for 2 patients: in 1 patient, the screen IBI result was recorded as highly positive, but a reagent issue prevented an exact IBI calculation; the repeated IBI at a dilution of 1:1000 was noted to be 29.3, consistent with a highly positive result. In the other patient, who had a screen IBI value of 33.3, there was insufficient quantity of sample remaining to determine the final end-titer result.

^b^ Longer than 3 contiguous vertebral body segments.

^c^ Radiologically isolated syndrome, clinically isolated syndrome, and multiple sclerosis with typical multiple sclerosis demyelinating lesions on brain magnetic resonance image.^9^

^d^ Other neurologic disorders variably included the following categories of suspected diagnoses at the time of MOG-IgG1 testing: (1) progressive optic neuropathy, myelopathy, or encephalomyelopathy/cognitive decline; (2) other acute encephalopathies without magnetic resonance imaging findings consistent with demyelination (eg, isolated limbic or metabolic encephalopathies); (3) clear stroke or vasculitic brain syndromes; (4) isolated headache or meningitis; (5) neuromuscular disorders; or (6) neurodegenerative disorders (eg, primary lateral sclerosis, hereditary spastic paraparesis).

^e^ The 2 individuals with true-positive MOGAD cases in this category had progressive optic neuropathy and encephalopathy, respectively, that presented insidiously, reaching the nadir beyond 1 month.

### True-Positive and False-Positive Results

**Figure 2.**
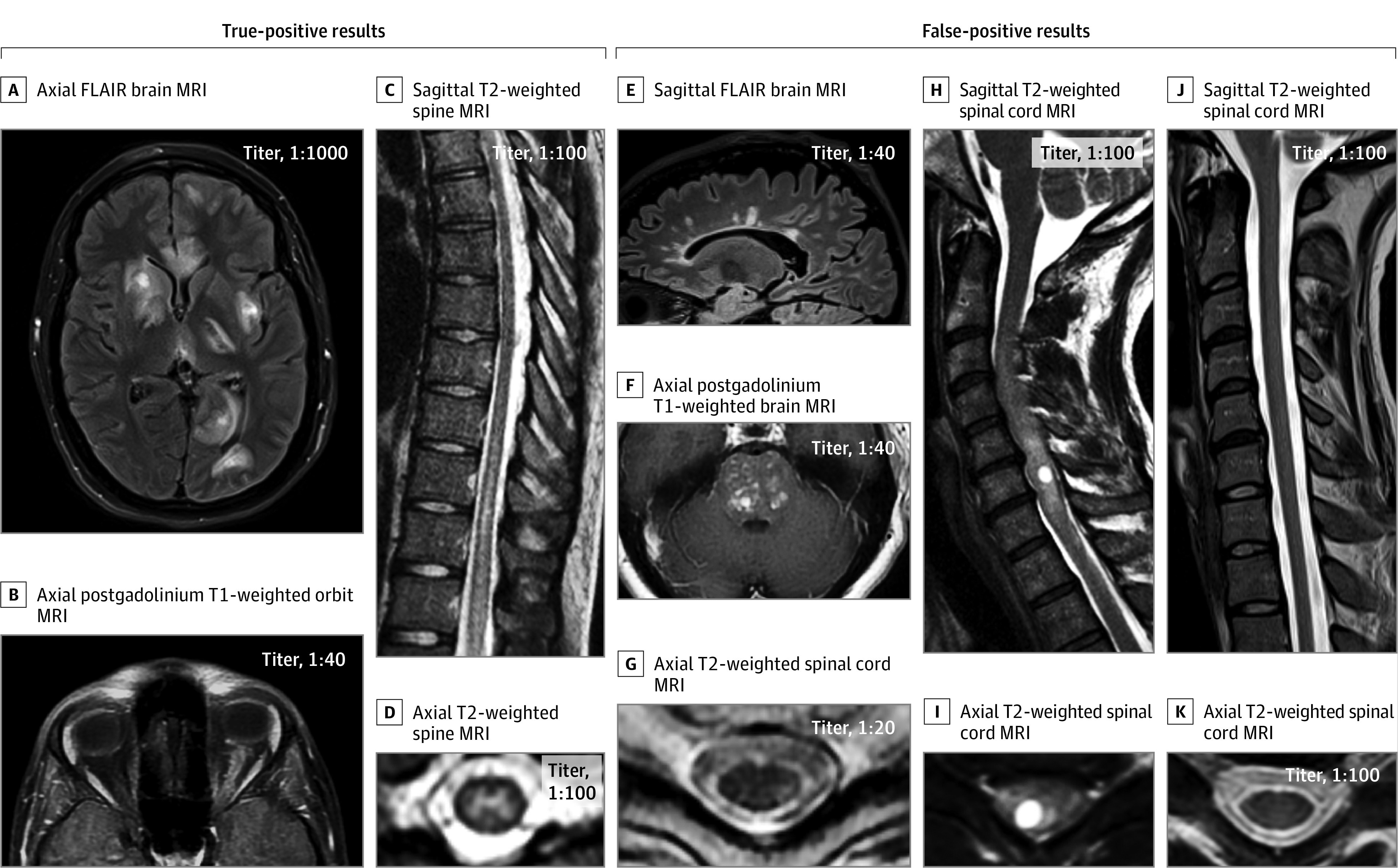
Representative Examples of Magnetic Resonance Imaging (MRI) Findings in True-Positive vs False-Positive Myelin Oligodendrocyte Glycoprotein (MOG)–IgG1 Cases True-positive cases (A-D): axial fluid-attenuated inversion recovery (FLAIR) image showing large, multifocal, poorly demarcated lesions on brain MRI in a patient with an acute disseminated encephalomyelitis attack in MOG-IgG1–associated disorder (MOGAD) (A); axial postgadolinium T1-weighted orbit MRI showing longitudinally extensive enhancement of the left optic nerve sheath in a patient with optic neuritis as a manifestation of MOGAD (B); sagittal T2-weighted images showing a longitudinally extensive myelitis lesion along the lower thoracic spinal cord, with predominant involvement of the central gray-matter on axial images in MOGAD (C and D). False-positive cases (E-K): sagittal FLAIR image showing Dawson-finger T2-hyperintense lesions perpendicular to the ventricle, typical of multiple sclerosis (E); axial postgadolinium T1-weighted images showing multiple areas of nodular enhancement in the pons that brainstem biopsy confirmed to be a histiocytic disorder (F); axial T2-weighted image showing a peripheral dorsolateral hyperintense lesion abutting the surface of the spinal cord, in another patient with multiple sclerosis (G); sagittal T2-weighted image showing a faint longitudinally extensive T2-hyperintense lesion accompanied by marked cervical spinal cord swelling with an intralesional cyst (H), also appreciable on axial images (I), which biopsy confirmed to be a glioma; sagittal (J) and axial (K) T2-weighted spinal cord MRI showing normal signal intensity and initial atrophy in a young adult man with X-linked adrenomyeloneuropathy.

The 2 independent raters agreed on 91 of 92 cases (99%) for true and false positivity. The single discordant case, with steroid-responsive subacute progressive encephalitis and brain biopsy results showing demyelination, remyelination, and perivascular lymphocytic inflammation, was designated as having a true-positive result after consensus. Twenty-six patients (28%) had false-positive results, including 11 with typical MS, 3 with infarction (1 in the optic nerve, 1 in the brainstem, and 1 in the spinal cord); 2 with B_12_ deficiency, 2 with biopsy-proven neoplasia (1 with a histiocytic neoplasia and 1 with glioma), 1 with genetically confirmed adrenomyeloneuropathy, 1 with an isolated caudate lesion (which was noninflammatory on biopsy), 1 with postradiation myelopathy, 1 with varicella-zostervirus–associated myelitis, 1 with probable neurosarcoidosis, 1 with idiopathic progressive cerebellar degeneration, 1 with idiopathic progressive myelopathy, and 1 with a nonneurologic syndrome. The median antibody titer was higher with true-positive results (1:100 [range, 1:20-1:10 000]) vs false-positive results (1:40 [range, 1:20-1:100]; *P* < .001). Cerebrospinal fluid–restricted oligoclonal bands were less common among those with true-positive results (2 of 49 [4%]) than those with false-positive results (8 of 18 [44%]; *P* < .001), and patients with true-negative results (265 of 790 [33.5%]; *P* < .01). Figure 2 shows representative MRIs of true-positive and false-positive cases.

## Discussion

This study confirms MOG-IgG1 as a highly specific biomarker of MOGAD in clinical practice. The increasing PPV with higher titers and pretest probability, and excellent agreement of independent raters on MOGAD phenotypes supports it being a distinct disease.^2^ However, more than one-quarter of positive results might be false in a high-throughput setting, and given the frequency of MOG-IgG1 testing requests (~20 000 samples/year in our laboratory), clinicians should be aware. Indiscriminate MOG-IgG1 testing is not recommended, and caution is advised when interpreting low-titer positivity with atypical phenotypes.

While the absolute requirement of MOG-IgG1 positivity for MOGAD diagnosis hinders sensitivity calculation, identification of alternative diagnoses or incompatible clinical-MRI phenotypes allows recognition of false-positive results for specificity and PPV calculation. Our specificity was similar to those of prior studies (97.8% vs 99.6%-100%^5,6^) that used experimental populations, but PPV in clinical practice was lower (72% vs 95.5%-100%^5,6^) because it was affected by disease prevalence and ordering practices.^2^ Increasing the MOG-IgG1 IBI screening cutoff value would increase the PPV but exclude many true MOGAD cases, and stratifying results by low (1:20-1:40) and high (≥1:100) titers might be preferred.

Multiple sclerosis was overrepresented among patients with false-positive results, which should dissuade clinicians from uniform ordering of MOG-IgG1 testing in patients with typical MS.^9^ Prior studies^12,13,14^ have shown a lower frequency of MOG-IgG1 positivity via live cell–based assays among patients with MS (0%-2% vs 2.5% in our study), possibly from differences in assay cutoff values, inclusion criteria, or referral bias. In clinical practice, MOG-IgG1 positivity requires careful evaluation for typical MOGAD clinical-MRI characteristics and red flags that argue against MOGAD (eg, a progressive course).^2^

Our findings have major implications for future updates of international consensus diagnostic criteria in MOGAD. More stringent clinical, radiologic, and laboratory requirements accompanying MOG-IgG1 positivity would help prevent misdiagnosis, inappropriate treatment, and enrollment of individuals with false-positive results in clinical trials that could hinder their success.

### Limitations

Our study has limitations. We did not evaluate cerebrospinal fluid, in which isolated MOG-IgG1 positivity may occur,^15^ but serum testing is generally recommended.^2^ The PPV observed in this study depends on the titer cutoff value used for MOG-IgG1 positivity and selection of different cutoff values would yield different PPVs. The PPV is also strongly associated with the population tested and ordering practices, and in other populations, in which MOGAD is less represented, the PPV could be lower. Similar to diagnostic criteria for other CNS-demyelinating diseases, the definition of true MOGAD was supported by exclusion of alternative diagnoses, and future identification of new CNS-demyelinating syndromes might reduce the PPV over time. Positivity for MOG-IgG1 was not confirmed in a second laboratory, but our assay is comparable with those of other centers,^5,6^ and repeated testing in clinical practice may not be available or practical, still requiring careful assessment for MOGAD phenotypes.

## Conclusions

Assessment of MOG-IgG1 by live cell-based assay is highly specific for MOGAD diagnosis but has a potential risk of false-positive results when tested indiscriminately in clinical practice. Future multicenter efforts should focus on assay improvements that could reduce false-positive results.

## Notes

Supplement. **eFigure.** Flowchart Showing Consecutive Steps for Inclusion of Mayo Clinic Patients in the Study
