# Supplementary material for: Positive Predictive Value of Myelin Oligodendrocyte Glycoprotein Autoantibody Testing
Source: JAMA Neurol. 2021 Apr 26;78(6):1–6. doi: 10.1001/jamaneurol.2021.0912 (PMC8077043; doi:10.1001/jamaneurol.2021.0912)
Supplement: Supplement. — eFigure. Flowchart Showing Consecutive Steps for Inclusion of Mayo Clinic Patients in the Study [file jamaneurol-e210912-s001.pdf]

Supplemental Online Content

Sechi E, Buciuc M, Pittock SJ, et al. Positive predictive value of myelin oligodendrocyte glycoprotein autoantibody testing. *JAMA Neurol*. Published online April 26, 2021.
doi:10.1001/jamaneurol.2021.0912

eFigure. Flow chart showing consecutive steps for inclusion of Mayo Clinic patients in the study

This supplemental material has been provided by the authors to give readers additional information about their work.

© 2021 American Medical Association. All rights reserved.

eFigure. Flow chart showing consecutive steps for inclusion of Mayo Clinic patients in the study

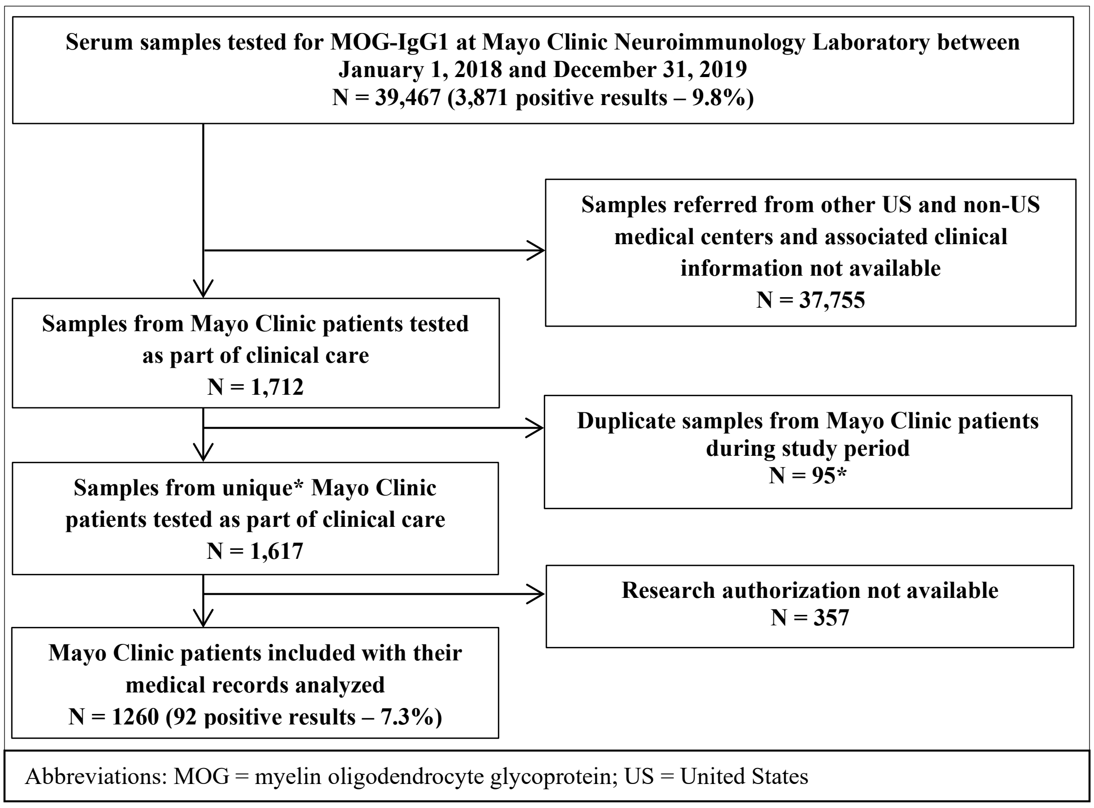

*MOG-IgG1 negative, 70; MOG-IgG1 positive, 25;

In those with multiple samples, the first positive result was used for analysis

© 2021 American Medical Association. All rights reserved.
